# The development of the PROMPT (PRescribing Optimally in Middle-aged People’s Treatments) criteria

**DOI:** 10.1186/s12913-014-0484-6

**Published:** 2014-10-30

**Authors:** Janine A Cooper, Cristín Ryan, Susan M Smith, Emma Wallace, Kathleen Bennett, Caitriona Cahir, David Williams, Mary Teeling, Tom Fahey, Carmel M Hughes, (The PROMPT Steering Group)

**Affiliations:** Clinical and Practice Research Group, School of Pharmacy, Queen’s University Belfast, 97 Lisburn Road, Belfast, Northern Ireland BT9 7BL UK; HRB Centre for Primary Care Research, Department of General Practice, Royal College of Surgeons in Ireland, 123 St Stephen’s Green, Dublin 2, Ireland; Department of Pharmacology & Therapeutics, Trinity Centre for Health Sciences, St James’s Hospital, Dublin 8, Ireland; Department of Geriatric and Stroke Medicine, Royal College of Surgeons in Ireland, 123 St Stephen’s Green, Dublin 2, Ireland

**Keywords:** Potentially inappropriate prescribing, Explicit criteria, Delphi technique, Middle-age, Polypharmacy, Multimorbidity

## Abstract

**Background:**

Whilst multimorbidity is more prevalent with increasing age, approximately 30% of middle-aged adults (45–64 years) are also affected. Several prescribing criteria have been developed to optimise medication use in older people (≥65 years) with little focus on potentially inappropriate prescribing (PIP) in middle-aged adults. We have developed a set of explicit prescribing criteria called PROMPT (PRescribing Optimally in Middle-aged People’s Treatments) which may be applied to prescribing datasets to determine the prevalence of PIP in this age-group.

**Methods:**

A literature search was conducted to identify published prescribing criteria for all age groups, with the Project Steering Group (convened for this study) adding further criteria for consideration, all of which were reviewed for relevance to middle-aged adults. These criteria underwent a two-round Delphi process, using an expert panel consisting of general practitioners, pharmacists and clinical pharmacologists from the United Kingdom and Republic of Ireland. Using web-based questionnaires, 17 panellists were asked to indicate their level of agreement with each criterion via a 5-point Likert scale (1 = Strongly Disagree, 5 = Strongly Agree) to assess the applicability to middle-aged adults in the absence of clinical information. Criteria were accepted/rejected/revised dependent on the panel’s level of agreement using the median response/interquartile range and additional comments.

**Results:**

Thirty-four criteria were rated in the first round of this exercise and consensus was achieved on 17 criteria which were accepted into the PROMPT criteria. Consensus was not reached on the remaining 17, and six criteria were removed following a review of the additional comments. The second round of this exercise focused on the remaining 11 criteria, some of which were revised following the first exercise. Five criteria were accepted from the second round, providing a final list of 22 criteria [gastro-intestinal system (n = 3), cardiovascular system (n = 4), respiratory system (n = 4), central nervous system (n = 6), infections (n = 1), endocrine system (n = 1), musculoskeletal system (n = 2), duplicates (n = 1)].

**Conclusions:**

PROMPT is the first set of prescribing criteria developed for use in middle-aged adults. The utility of these criteria will be tested in future studies using prescribing datasets.

## Background

Although a universal definition has yet to be adopted, polypharmacy has conventionally been described as the use of four or more medications [1]. A principal factor contributing to polypharmacy is multimorbidity, which is usually defined as the presence of two or more long-term conditions [2]. As most long-term conditions are managed by reference to separate evidence-based guidelines, the lack of guidance on prioritising treatments in patients with multiple conditions can lead to polypharmacy and is a challenge for healthcare professionals [3]. One difficulty in the prescribing for multimorbid patients is the risk of potentially inappropriate prescribing (PIP), described as the use of treatments which increase the risk of harm to a patient, or where a similar, or more effective alternative is available which has a lower risk to the patient [4]. Conceptually, inappropriate prescribing encompasses a range of behaviours for healthcare providers, including errors in prescribing, over-prescribing, under-prescribing, cost effectiveness, non-adherence and alternative therapies [5].

Traditionally, the focus of PIP has been on older people (defined as aged 65 years and above) due to the high prevalence of medication use in this age group and age-related changes in pharmacokinetics and pharmacodynamics [6,7]. However, there is evidence that multimorbidity is also prevalent in middle-aged people (defined as aged between 45 and 64 years) [2], but as yet, there has been little consideration of PIP in this age group.

Prescribing tools have been developed to identify PIP and may contain explicit or implicit criteria. Implicit criteria are patient-specific and are designed for use with clinical knowledge and judgement, but may be time-consuming to use [7,8]. Explicit criteria are developed from literature reviews, expert opinion and consensus, require little or no clinical judgement, and can be applied to prescribing datasets in the absence of clinical information [7,8]. However, explicit criteria need regular updates and may require adaption for use in other countries dependent on local guidelines and accepted clinical practice [5]. Several explicit prescribing criteria have been developed for application in older people to examine the prevalence of PIP and optimise prescribing [9-16]. Explicit criteria have proven effectiveness in determining the prevalence of PIP using population-based data; for example, using a subset of the STOPP (Screening Tool of Older Person’s Prescriptions) and START (Screening Tool to Alert doctors to Right Treatment) screening tools, studies have shown an overall prevalence of PIP of 34% in Northern Ireland and 36% in the Republic of Ireland among older people [17,18].

Published prescribing criteria for use in older people often consist of a large number of criteria to be considered, and many of these criteria would be redundant in people aged between 45–64 years due to the differences in the prevalence of diseases and drugs used in these patient groups, and changes in pharmacokinetics and drug metabolism associated with ageing [6,7]. We have identified the need for a prescribing tool specifically for the middle-aged population, containing criteria which are relevant for this age-group. Such a tool may be used to determine the prevalence of PIP in middle-aged people and provide a quick and easy-to-use resource for healthcare professionals. For this reason, the present study aimed to develop the first set of explicit prescribing criteria for middle-aged people called PROMPT (PRescribing Optimally in Middle-aged People’s Treatments) which could be applied to prescribing datasets, independent of clinical information, to determine the prevalence of PIP in middle-aged people.

## Methods

### Study design

A Delphi consensus technique was used to develop these criteria. Delphi consensus allows an estimate of an overall group opinion to be reached by improving agreement between a panel of experts through rounds of questionnaires [19]. This process has been successful in the development of previous explicit prescribing criteria for older people [9-11,14]. Ethical approval for this study was obtained from the School of Pharmacy, Queen’s University Belfast.

### Compilation of initial criteria

**Figure 1:**
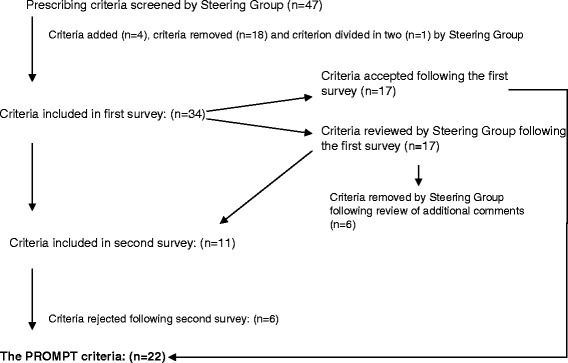
**A flow chart for the development of the PROMPT criteria.**

The process used to develop the PROMPT criteria is outlined in Figure 1. We did not seek to undertake a systematic review of the literature relating to prescribing criteria in older people. In order to inform the development of PROMPT, commonly used prescribing criteria relating to PIP in older people were identified by the Project Steering Group (shown in Table 1). Using these sets of published criteria, three members of the Project Steering Group (J.C., C.R. and C.H.) primarily considered the relevance of each criterion to the target population. Criteria containing conditions which are generally uncommon in middle-aged adults (e.g. dementia) or containing medications currently unavailable in the United Kingdom or the Republic of Ireland were excluded. Criteria which could not be applied in the absence of clinical information were also excluded. Following these exclusions, a truncated list was reviewed by all members of the Project Steering Group by a consensus discussion. Members added further criteria for consideration that arose during group discussion based on the clinical experience and expertise of the project steering group, for example ‘mucolytic agents (e.g. carbocisteine, mecysteine) should not be used routinely in stable chronic obstructive pulmonary disease’. In this process, the Project Steering Group considered many factors, including applicability of the criterion in the absence of clinical information, prevalence of drug use and the rationale for using a criterion in middle-aged adults e.g. clinical relevance. Finally, the Project Steering Group also reviewed the prevalence of individual drug use in middle-aged adults using dispensing data from the Enhanced Prescribing Database (EPD) and the Primary Care Reimbursement Service (PCRS). The EPD is a population-based dataset which stores information on computer-generated prescriptions, issued in general practice which have been subsequently dispensed by a community pharmacy in Northern Ireland. The PCRS stores information on all medications, and other health services, provided without charge to people eligible for free medical services via means testing and therefore is not representative of the entire population of Ireland. Potential criteria containing medications with a low prevalence (i.e. to define uncommon use, a cut-off of less than 0.5% was agreed by the Project Steering Group) were excluded. Criteria were also excluded if they were not applicable in the absence of clinical information. For example, the criterion *‘Patients with heart failure receiving a potassium sparing diuretic (spironolactone) should not be prescribed a: non-steroidal anti-inflammatory drug (NSAIDs), angiotensin converting-enzyme inhibitor (ACE), angiotensin-II receptor antagonist (ARB) or potassium supplement’* and the accompanying rationale *‘In patients with heart failure, the risk of hyperkalaemia is higher’* was removed by the Project Steering Group during this screening stage as the criterion related specifically to patients with heart failure and could only be successfully applied to a dataset with clinical information. However, the Project Steering Group retained some criteria which included reference to a specific clinical diagnosis as it was still possible to apply these criteria to prescribing data without clinical information. For example, in the criterion *‘Theophylline should not be used as monotherapy for asthma or chronic obstructive pulmonary disease’*, a prescription for an inhaled beta agonist, corticosteroid or muscarinic antagonist, a leukotriene receptor antagonist or theophylline may be used as a proxy for other therapy for asthma and COPD. Finally, some criteria identified from literature were modified by the Project Steering Group to make them applicable to dispensing data (for example, we defined long-term use as greater than three months’ dispensed medicine).

**Table 1:** **Criteria screened for inclusion in PROMPT**

**Criteria name**	**Origin**	**Year**	**Method**
Beers Criteria [9]	United States of America	2012 update	Modified Delphi method
The PRISCUS List [10]	Germany	2010	Delphi consensus technique
NORGEP Norwegian General Practice Criteria [11]	Norway	2009	Delphi consensus technique
Basger Criteria [12]	Australia	2008	Prescribing prevalence and review of drug information
Winit-Watjana Criteria [13]	Thailand	2008	Delphi consensus technique
STOPP Screening Tool for Older Person’s Prescriptions [14]	Republic of Ireland	2008	Delphi consensus technique
START Screening Tool to Alert doctors to Right Treatment[14]	Republic of Ireland	2008	Delphi consensus technique
Laroche Criteria [15]	France	2007	Delphi consensus technique
McLeod Criteria [16]	Canada	1997	Delphi consensus technique

### Selection of the Delphi panel

Thirty specialists from the United Kingdom and Republic of Ireland, recognised as experts in their fields, were invited *a priori* (via e-mail) to participate in a Delphi consensus panel to develop these criteria. Reasons for non-participation by the experts were not sought, however the most common explanation for a refusal was due to lack of time. Of the 30 experts (who comprised experienced academic GPs, academic/clinical pharmacists and clinical pharmacologists/physicians, identified by the Project Steering Group) invited to join the panel, 17 agreed to participate, and were representative of all specialists invited to participate in terms of location and expertise. Consent was received from all participating panel members before commencing the process.

### Data collection and analyses

The consensus process involved two rounds of web-based questionnaires. The first questionnaire was piloted (to test usability) by two members of staff in the School of Pharmacy, Queen’s University of Belfast and modified accordingly. The first and second rounds of this development process took place between July 2013 and September 2013, and between October 2013 and November 2013, respectively. For each of these rounds, panel members received a link (via e-mail) to a questionnaire which was maintained on an online survey software tool (SurveyGizmo®). Reminders were sent to all panellists via e-mail to encourage completion of the exercise. The aims of the study were explained to the panel members in the email, who were asked to assess the applicability of each criterion to middle-aged adults in the absence of clinical information. Panellists were presented with statements and accompanying rationales, categorised by physiological systems (gastro-intestinal system, cardiovascular system, respiratory system, central nervous system, infections, endocrine system and musculoskeletal system) and a category for duplicate drug classes. Panellists were asked to indicate their level of agreement with each statement using a 5-point Likert scale [20] (where 1 was strongly disagree and 5 was strongly agree) and to provide comments as necessary. Using this scale, the median response and the interquartile range were calculated and the level required for consensus between the panel members was decided prior to commencing the study. When the upper quartile was ≤2, this indicated there was general disagreement with the criterion between the panel members, and the criterion was rejected. When the lower quartile was ≥4, this indicated there was general agreement with the criterion between the panel members, and the criterion was accepted. When the interquartile range included 3, this indicated there was a lack of agreement between the panel members and a need for further review of the particular criterion. Where the interquartile range included 3, criteria were reviewed by Project Steering Group (via discussion) and were either revised and included in the second questionnaire or rejected based on the additional comments received from the panel members. Panellists did not receive feedback from the first questionnaire. In the second questionnaire, panellists were provided with a link to the most recent guidelines supporting each criterion. As before, the median response and the interquartile range were calculated, and these measures of agreement (along with any additional comments) were reviewed by Project Steering Group. If consensus was not reached following the second survey, the criterion (or part of the criterion in which there was still disagreement) was rejected on the basis of the median response/interquartile range and the comments provided by the panel members.

## Results

In total, 47 published prescribing criteria for older people were screened by the Project Steering Group. Eighteen criteria were removed by the Steering Group subject to the pre-defined exclusion factors, four criteria were added based on their own clinical experience and one criterion was further divided into two criteria. Thirty-four criteria were presented in the first round of this development process, and all 17 panel members who agreed to participate in this Delphi exercise completed the questionnaire. Group consensus (by overall agreement) was achieved for 17 criteria, which were accepted into the PROMPT criteria (Table 2). No prescribing criteria were rejected following the first round on level of agreement between Delphi panel members. However, consensus was not reached on the remaining 17 criteria. From these 17 criteria, 6 criteria were rejected by the Project Steering Group following a review of the respondents’ additional comments, leaving 11 criteria for consideration in the second round of the Delphi consensus exercise. The main reasons for exclusion of criteria were the perceived need for clinical information, drug or drug classes which were rarely seen in practice by the panel members, lack of consensus and non-applicability to datasets.

**Table 2:** **Outcomes from Delphi consensus exercises**

	**First round**				**Steering group consensus**	**Second round**				**Final set of criteria**
**Section**	**Total**	**Accepted**	Revision*	**Rejected**	**Removal following first round**	**Total**	**Accepted**	Revision* ^**¶**^	**Rejected**	**Total accepted**
**Gastro-intestinal system**	4	1	3	0	1	2	1	1	-	3
**Cardiovascular system**	8	4	4	0	1	3	-	-	3	4
**Respiratory system**	4	3	1	0	0	1	1	-	-	4
**Central nervous system**	10	5	5	0	2	3	-	1	2	6
**Infections**	2	1	1	0	1	-	-	-	-	1
**Endocrine system**	2	1	1	0	1	-	-	-	-	1
**Musculoskeletal system**	3	1	2	0	0	2^§^	-	1	1	2
**Duplicates**	1	1	0	0	0	-	-	-	-	1
**Total**	34	17	17	0	6	11	2	3	6	22

*Required revision, rewording or refinement ^¶^Required removal of part of criterion.

^§^The Project Steering Group moved one criterion to another section after the first round, this criterion was excluded in round two (changes not shown in table).

The second round of this developmental exercise was completed by 15 of the 17 panel members who agreed to participate. We were unable to identify which members had left the panel due to the anonymity of this exercise, and therefore the reasons for non-participation in the second round could not be obtained. Of the 11 criteria in the second questionnaire, consensus was reached in five criteria which were accepted into the PROMPT criteria. The remaining six criteria were rejected following a review of the level of agreement and additional comments from the panel members. The main reasons for exclusion of criteria following the second round were the need for clinical information and lack of consensus among panel members. Some exemplar comments received from the Delphi Panel are shown in Table 3.

**Table 3:** **Exemplar comments received from the Delphi panel**

**Section**	**Gastro-intestinal section**	**Respiratory section**
**Example of statement used in Round 1**	Stimulant laxatives (e.g. bisacodyl, senna) should not be used long-term i.e. for greater than four weeks.	First generation antihistamines (e.g. chlorphenamine, promethazine) should not be used for greater than seven days.
	Rationale: Stimulant laxatives are not suitable for long-term use (greater than four weeks), due to risk of dependency and decreased bowel function.	Rationale: First generation antihistamines exert anticholinergic properties causing unwanted side-effects e.g. constipation, drowsiness, psychomotor impairment.
Comments from Round 1*	C1: *Chronic management sometimes required.*	C1: *Addiction is a problem with these agents.*
	C4: *Regular Prescribing often led by patient demand.*	C3: *Depends on indication, alternatives tried and their effect. Certainly not first line.*
	C5: *Lack of evidence base regarding effect on long term bowel function, old case reports likely consequent to adulteration in laxative preparation.*	C4: *Often led by patient demand. but most likely will be on 2nd generation antihistamine.*
**Revisions made for Round 2 (revisions shown in bold text)**	Stimulant laxatives (e.g. bisacodyl, senna) should not be prescribed **as first-line treatment in constipation** for greater than four weeks **(other than for opioid induced constipation).**	First generation antihistamines (e.g. chlorphenamine, promethazine) should not be used **as first-line agents** for greater than seven days.
	Rationale: Stimulant laxatives are not suitable for continuous long-term use, **other than for opioid induced constipation.**	Rationale: First generation antihistamines **may cause addiction** and/or exert anticholinergic properties causing unwanted side-effects e.g. constipation, drowsiness, psychomotor impairment.
Comments from Round 2*	C1: *Stimulants are only licenced for short term use, but the guidance you steer us to does not say anything that comes close to the indicator in terms of “should not be prescribed”, so I don’t think it’s a sensible indicator (I’d be happier to support one about “should use” stimulants in people on strong opioids).*	C1: *You would only continue using them if the patient either didn’t respond to other antihistamines and/or didn’t have any of the above side effects- I have seen plenty of patients who are fully function on full dose chlorphenamine. Of course, they can buy it OTC so I guess there are lots of people out there who (we hope) are fine and using it.*
	C2: *However we recognise that some patients will buy these products and/or may be using them without health professional knowledge or advice. Best practice is to review after four weeks and reassess for alternatives.*	C2: *Especially for hypnotic indications.*
	C3: *Bulk forming or osmotic laxatives should be used first.*	
**Conclusion**	Further rewording following Round 2. Final statement:	No further revision following Round 2. Final statement:
	Other than for opioid-induced constipation, stimulant laxatives (e.g. bisacodyl, senna) should not be prescribed as first-line treatment in constipation for greater than four weeks.	First generation antihistamines (e.g. chlorphenamine, promethazine) should not be used as first-line agents for greater than seven days.
	Rationale: Stimulant laxatives are not suitable for continuous long-term use, other than for opioid induced constipation.	Rationale: First generation antihistamines may cause addiction and/or exert anticholinergic properties causing unwanted side-effects e.g. constipation, drowsiness, psychomotor impairment.

*Please note, this is only a selection of comments used in the revision of exemplar criteria to convey the views of the Panel and how criteria were subsequently revised.

The final PROMPT criteria consisted of 22 criteria organised over the following physiological systems: Gastro-Intestinal System (n = 3), Cardiovascular System (n = 4), Respiratory System (n = 4), Central Nervous System (n = 6), Infections (n = 1), Endocrine System (n = 1), Musculoskeletal System (n = 2), Duplicates (n = 1). The final set of criteria is presented Table 4.

**Table 4:** **The PROMPT criteria**

**Original source(s) for criteria**	**Section**	**Rationale**
	**Gastro-Intestinal System**	
[15]	**Other than for opioid-induced constipation, stimulant laxatives (e.g. bisacodyl, senna) should not be prescribed as first-line treatment in constipation for greater than four weeks.**	*Stimulant laxatives are not suitable for continuous long-term use, other than for opioid induced constipation.*
[14]	**Proton pump inhibitors (PPIs) (e.g. esomeprazole, omeprazole) should not be prescribed at doses above the recommended maintenance dosage for greater than eight weeks.**	*A dose reduction or discontinuation is indicated since there is no therapeutic benefit observed with the use of higher doses of PPIs long-term (unless treatment is indicated for rare conditions e.g. Zollinger-Ellison syndrome).*
*Added by Project Steering Group* [21]	**Esomeprazole or omeprazole should not be used in combination with clopidogrel.**	*Esomeprazole and omeprazole may reduce the anti-platelet effect of clopidogrel and therefore should not be used in combination with clopidogrel. Other proton pump inhibitors or H*_*2*_ *-receptor antagonists are available which do not have the same potential for interaction.*
	**Cardiovascular System**	
[9,10,13]	**The use of alpha-adrenoceptor blocking drugs (e.g. doxazosin, prazosin) as monotherapy for hypertension, should be avoided.**	*Alpha-adrenoceptor blocking drugs increase the risk of orthostatic hypotension.*
[14]	**Aspirin doses should not exceed 150 mg/day for anti-platelet therapy.**	*Doses exceeding 150 mg/day show no evidence for increased efficacy and will increase the risk of bleeding.*
[11,14]	**Cardio-selective calcium-channel blockers (e.g. verapamil, diltiazem) should not be used in combination with beta-adrenoceptor blocking drugs.**	*Concomitant use increases the risk of atrioventricular block and myocardial depression.*
[9,14-16]	**The use of oral short-acting dipyridamole should not be used as monotherapy in antiplatelet treatment.**	*Oral short-acting dipyridamole may cause orthostatic hypotension; more effective alternatives available.*
	**Respiratory System**	
[9-11,14,15]	**First generation antihistamines (e.g. chlorphenamine, promethazine) should not be used as first-line agents for greater than seven days.**	*First generation antihistamines may cause addiction and/or exert anticholinergic properties causing unwanted side-effects e.g. constipation, drowsiness, psychomotor impairment.*
[11,14]	**Theophylline should not be used as monotherapy for asthma or chronic obstructive pulmonary disease.**	Theophylline is associated with an increased risk of arrhythmias.
[14]	**A concomitant bisphosphonate should be prescribed if oral corticosteroids are used long-term (greater than three months).**	*Long-term use of an oral corticosteroid increases the risk of osteoporosis and subsequent bone fracture.*
*Added by Project Steering Group* [22]	**Mucolytic agents (e.g. carbocisteine, mecysteine) should not be used routinely in stable chronic obstructive pulmonary disease.**	*There is little benefit from the use of mucolytic agents in stable chronic obstructive pulmonary disease.*
	**Central Nervous System**	
[12]	**Selective serotonin reuptake inhibitors (e.g. citalopram, fluoxetine) should not be used in combination with venlafaxine.**	*Concomitant use may lead to the development of serotonin syndrome.*
[9-13,15]	**Tricyclic antidepressants (TCAs) (e.g. amitriptyline, nortriptyline) should not be used as first-line in treatment of depression.**	*TCAs are associated with unwanted peripheral anticholinergic side-effects e.g. constipation, dry mouth and central anticholinergic side-effects e.g. drowsiness.*
[9-16]	**Benzodiazepines (e.g. nitrazepam, temazepam) should not be used long-term (greater than four weeks).**	*Long-term use of benzodiazepines increases the risk of dependency. Benzodiazepine related adverse effects include daytime sedation, cognitive impairment, agitation, irritability.*
[9,10]	**Non-benzodiazepine hypnotics (zolpidem, zaleplon, zopiclone) should not be used long-term (greater than 4 weeks).**	*Non-benzodiazepine hypnotics have adverse events similar to those of benzodiazepines with minimal improvement in sleep latency and duration.*
[11]	**Carbamazepine should not be used in combination with clarithromycin or erythromycin.**	*Clarithromycin and erythromycin inhibit the metabolism of carbamazepine therefore increasing the risk of adverse effects e.g. headache, drowsiness, nausea.*
[12,14]	**Strong opioids (e.g. buprenorphine, diamorphine, fentanyl, morphine, oxycodone) should not be prescribed without the co-prescribing of laxatives.**	*Strong opioids are likely to cause constipation.*
	**Infections**	
[9,10,15]	**Nitrofurantoin should not be prescribed for greater than 7 days for the management of uncomplicated lower urinary-tract infections.**	*Potential for pulmonary toxicity; safer alternatives available.*
	**Endocrine System**	
[9,14,15]	**In relation to the management of diabetes, the use of oral long-acting sulfonylureas (glibenclamide) should be avoided.**	*Oral long-acting sulfonylureas have a prolonged half-life and can cause prolonged hypoglycaemia or syndrome of inappropriate antidiuretic hormone (ADH) secretion.*
	**Musculoskeletal System**	
[9,10,13,14,16]	**Non-steroidal anti-inflammatory drugs (NSAIDs) (e.g. celecoxib, diclofenac, naproxen) should not be used long-term (greater than three months).**	*Long-term NSAID treatment should be reviewed periodically due to increased risk of thrombotic effects, and the lowest effective dose should be prescribed for the shortest period.*
[11-13]	**Unless adequate gastro-intestinal protection is provided with either a proton pump inhibitor or H**_**2**_ **-receptor antagonist, non-steroidal anti-inflammatory drugs should not be used in combination with:**	*Concomitant use increases the risk of gastro-intestinal bleeding.*
	**a. Low-dose aspirin.**	
	**b. Selective serotonin re-uptake inhibitors.**	
	**Duplication of drug classes**	
[11,12,14,15]	**The use of two or more drugs from the same pharmacological class should be avoided, unless used for additive effects in line with current clinical guidelines.**	*Possible unwanted duplication of effect, increasing risk of side effects and adverse events.*
	**For example: Avoid duplication of opioid analgesics, non-steroidal anti-inflammatory drugs, benzodiazepines.**	*An example of an exception includes: duplicate beta*_*2*_ *agonists (provided one is short-acting and one is long-acting) for the management of asthma or chronic obstructive pulmonary disease.*

## Discussion

Using a Delphi consensus method, we have developed a consensus-based set of prescribing criteria specifically for use in middle-aged adults. PROMPT consists of 22 statements relating to potentially inappropriate use of medications and has been developed for utilisation in prescribing or dispensing datasets in the absence of clinical information. The purpose of these criteria is for use in determining the prevalence of PIP in datasets containing information on dispensed medications (and may therefore serve as a proxy for prescribing quality) by adopting epidemiological approaches, but also for use as part of a screening approach in clinical practice. In primary care, healthcare professionals such as community pharmacists may not have access to medical records including diagnosis, and the PROMPT criteria may be a resource in this setting. The PROMPT criteria could also be used by pharmacists to perform medication reviews or to check dispensing queries when issuing medications to middle-aged adults with multimorbidity. Finally, as continuity of care has been highlighted as an issue for people living with multimorbidity who have higher consultation rates [23], coupled with the fragmentation of care, the PROMPT criteria may be used to identify areas where the greatest PIP burden lies and assist in targeting an intervention, such as a clinical decision support system (CDSS). With this in mind, PROMPT was not developed as an exhaustive list for PIP in middle aged adults. Instead, these criteria represent a list of commonly prescribed medications in the United Kingdom or the Republic of Ireland which may be used to explore the PIP burden, and factors associated with PIP such as age, gender and polypharmacy in this age group via descriptive studies using prescribing or dispensing databases. Following the development of these criteria, the utility and validity of PROMPT will be tested in future studies using national prescription-based databases. PROMPT may be used in other countries by researchers, but may require translation, and some modifications based on country specific prescribing guidelines, clinical practice and drug formularies [5].

Using the Delphi consensus method, the panel members informed the development of these criteria through their level of agreement and additional comments. Furthermore, the revisions to the criteria following each consensus round were based on comments received from the panel members. Some criteria were rejected as panel members were concerned that clinical information would be required to successfully apply the criteria. Other issues from panellists included difficulties in determining the appropriateness of a prescribed medication without knowledge of whether a treatment had been initiated by a specialist or if therapy was being correctly monitored (for example, via regular laboratory tests). Therefore, medications which may be prescribed primarily in specialty care settings have not been included. An attempt will be made to address these issues in future versions of the PROMPT criteria by incorporating clinical information and drug monitoring. Panel members were also concerned that although they may agree with a criterion in principle, they found that it did not fit with standard clinical practice or other influences. For example, in the criterion which stated *‘stimulant laxatives (e.g. bisacodyl, senna) should not be used long-term i.e. for greater than four weeks’* regular prescribing of some drug classes such as laxatives may be dictated by patient demand.

### Strength and limitations

This study has several key strengths. Firstly, the PROMPT criteria were constructed from two sources (clinical experience of the Project Steering group and from a review of published sets of criteria for older people) incorporating criteria across a range of physiological systems. Some criteria included in PROMPT were identified in many of the existing prescribing tools for older people (Table 1). However, the STOPP screening tool was the most commonly cited set of prescribing criteria, showing some overlap with these sets of criteria. Other criteria frequently cited in PROMPT were the Beers criteria (n = 9), Laroche criteria (n = 8) and NORGEP Norwegian General Practice criteria (n = 8) [9,11,15]. Second, references to prescribing guidelines were not provided in the first questionnaire, but were supplied for the second questionnaire, at the request of the Delphi panel. This allowed an informed decision (after considering relevant guidelines) to be made by the panellists for criteria where there was a lack of consensus. Third, the Delphi panel responsible for screening the criteria consisted of a heterogeneous group of experts, who all consented to participate prior to the first round of the questionnaire being distributed. Furthermore, this panel of experts represented a range of disciplines from geographically diverse areas of the United Kingdom and the Republic of Ireland, making the expert group representative of healthcare professionals involved in the care of our target population. Fourth, the number of rounds and consensus method was decided in advance of the questionnaire distribution, with criteria accepted or rejected following pre-defined instructions. Finally, panellists were not provided with feedback following each round of the development exercise, therefore removing any potential bias of panellists modifying their own responses to match those of the groups.

As with all developmental studies for PIP criteria which use a Delphi exercise, there are associated limitations. These include the potential lack of reproducibility as results may be dependent on the experts chosen for the Delphi panel [24]. We attempted to limit this potential bias by inviting a heterogeneous group of panel members to participate in this exercise. Other limitations include the lack of accountability for responses in the use of anonymous surveys and the lack of responses from panellists within increasing rounds which can cause bias [24]. It was intended that the use of anonymous surveys would mean panellists were not influenced by other members of the panel or the Project Steering Group. This did have a disadvantage, since the questionnaire responses could not be linked to any one individual, we were unable to identify members of the group who did not complete the second questionnaire. Therefore, a reminder was issued to all members of the Delphi panel to complete each round of the questionnaire. The second round questionnaire was completed by 15 of the 17 panel members who participated in the first round. However, this level of dropout among panellists is common in other developmental studies for PIP criteria [11]. Finally, these criteria will require future validation against healthcare outcomes such as hospitalisation or mortality.

### Comparison with existing literature

A recent review of published criteria has examined many aspects of inappropriate prescribing, including errors in prescribing, over-prescribing, under-prescribing, cost ineffectiveness, non-adherence and alternative therapies [5]. Most of the criteria included in PROMPT address PIP associated with the over-prescribing of medications in middle-aged adults. However, some criteria in PROMPT address under-prescribing or omissions. For example, ‘*A concomitant bisphosphonate should be prescribed if oral corticosteroids are used long-term (greater than three months)*’ with the supporting rationale *‘Long-term use of an oral corticosteroid increases the risk of osteoporosis and subsequent bone fracture*’. The START screening tool can be used to measure medication omissions in older age adults [14]. A future version of PROMPT which includes all aspects of PIP may provide further resources for assessing the burden of PIP in this age-group and support more comprehensive clinical medical reviews.

To date, there has been little consideration of PIP in middle-aged adults, although recent evidence has suggested that multimorbidity is prevalent in middle-aged people [2]. A recent cross-sectional study, which included over 1,750,000 people registered with 314 medical practices in Scotland, found that 30% of people aged between 45 and 64 years had multimorbidity [2]. This study highlighted the link between multimorbidity and increasing age, socioeconomic deprivation, mental health disorders (particularly depression) and gender [2]. The increasing prevalence of multimorbidity in middle-aged adults may also have economic implications. A retrospective observational study using data from the Clinical Practice Research Datalink (formerly known as the General Practice Research Database) investigated the burden of multimorbidity on primary care resources in a cohort of over 86,000 patients aged 20 years and over in the United Kingdom [25]. In all age groups, estimated prevalence-adjusted costs (including consultations, prescribed medication and tests) showed that patients living with any two conditions of depression, obesity, diabetes or asthma were associated with the greatest cost effect [25]. Considering this emerging evidence, studies aimed at determining the prevalence of PIP using the PROMPT criteria may identify important patterns in the prescribing of medications in middle-aged adults with multimorbidity and provide some evidence of the level of economic burden associated with PIP in this age-group. PROMPT may also identify areas to improve the quality of life in this age-group. A cross-border study in Ireland which analysed over 6000 adults aged 50 years and over [data from the 2007 Survey of Lifestyle, Attitudes and Nutrition in Ireland (SLAN) and the 2005 Northern Ireland Health and Social Wellbeing Survey (NIHSWS)] found that people living with multimorbidity were at the greatest risk of disability, poor self-rated health and reduced quality of life [26]. PROMPT may therefore have a role in investigating the independent effects between PIP, polypharmacy, multimorbidity and quality of life health outcomes in future quantitative and qualitative studies.

## Conclusions

Using a Delphi consensus method, we have developed the first set of prescribing criteria specifically for use in middle-aged adults. The PROMPT criteria may be applied to prescription-based datasets in the absence of clinical information to determine the prevalence of PIP, and the utility of these criteria will be tested in future studies using prescribing datasets.

## Abbreviations

ACE: Angiotensin converting-enzyme inhibitor
ARB: Angiotensin-II receptor antagonist
CDSS: Clinical decision support system
EPD: Enhanced prescribing database
NIHSWS: Northern Ireland health and social wellbeing survey
NSAID: Non-steroidal anti-inflammatory drug
PIP: Potentially inappropriate prescribing
PCRS: Primary care reimbursement service
PPI: Proton pump inhibitors
PROMPT: Prescribing optimally in middle-aged people’s treatments
SLAN: Survey of lifestyle, attitudes and nutrition in Ireland
STOPP: Screening tool of older person’s prescriptions
START: Screening tool to alert doctors to right treatment
TCA: Tricyclic antidepressant
